# Fat‐1 expression alleviates atherosclerosis in transgenic rabbits

**DOI:** 10.1111/jcmm.17188

**Published:** 2022-01-18

**Authors:** Chenyang Zhang, Xiaojing Wang, Suping Sun, Yu Fu, Yi Wu, Sihai Zhao, Xinzhong Fan, Enqi Liu

**Affiliations:** ^1^ Research Institute of Atherosclerotic Disease Xi’an Jiaotong University Cardiovascular Research Center Xi’an China; ^2^ Laboratory Animal Center Xi’an Jiaotong University Health Science Centre Xi’an China; ^3^ Department of Pathology Xi'an Central Hospital Xi’an China; ^4^ 34734 College of Animal Science and Veterinary Medicine Shandong Agricultural University Tai’an China; ^5^ School of Basic Medical Sciences Xi'an Jiaotong University Xi’an China

**Keywords:** atherosclerosis, *Fat‐1*, n‐3 polyunsaturated fatty acids, rabbit, transgenic

## Abstract

Atherosclerosis is the main cause of cardiovascular diseases. The *Fat*‐*1* gene can express the n‐3 fatty acid desaturase, which converts n‐6 polyunsaturated fatty acids (PUFA) to n‐3 PUFAs. The role of n‐3 PUFAs in atherosclerosis is widely debated. This study explored the effect of n‐3 PUFAs on atherosclerosis in rabbits. In this study, atherosclerosis was induced in *Fat*‐*1* transgenic rabbits and their littermate (WT) rabbits by feeding a high‐cholesterol diet containing 0.3% cholesterol and 3% soybean oil for 16 weeks. Plasma lipid, fatty acid and pathological analyses of atherosclerotic lesions were conducted. Fatty acid composition in the liver and muscle showed that n‐3 PUFAs increased and n‐6 PUFAs decreased in the Fat‐1 group. Plasma high‐density lipoprotein cholesterol (HDL‐C) levels were significantly increased in the Fat‐1 group, and the atherosclerotic lesion area of the aortic arch in *Fat*‐*1* transgenic rabbits was significantly reduced. Histological analysis showed that smooth muscle cells (SMCs) in atherosclerotic lesions decreased significantly. In conclusion, n‐3 PUFAs improve atherosclerosis in Fat‐1 transgenic rabbits, and this process may depend on the increase in plasma HDL‐C and the decrease in the amount of SMCs in atherosclerotic plaques.

## 1 INTRODUCTION

Atherosclerotic cardiovascular disease (CVD) is one of the main causes of morbidity and mortality globally, and causes severe economic and health damage.^1^, ^2^ Atherosclerosis is the build‐up of fatty or fibrous material in the innermost layer of an artery, leading to thickening of the arterial wall. A small number of lesions are produced in early atherosclerosis, which do not affect the circulation of blood flow in the body. However, with the gradual increase of plaque in the inner wall of the artery, the artery becomes narrow or there is extensive thrombus accumulation. At this time, sufficient blood cannot be provided to various organs, leading to the outbreak of CVD.^3^, ^4^ At present, several drugs are used to treat atherosclerotic CVD.^5^, ^6^ Due to the complexity of atherosclerosis, more drugs are needed to treat atherosclerosis.

n‐3 polyunsaturated fatty acids (n‐3 PUFAs) are the members of the PUFA family. PUFAs contain two or more double bonds and can be divided into n‐3 PUFA and n‐6 PUFA, according to the position of the double bonds. n‐3 PUFA refers to the unsaturated fatty acid on the third penultimate carbon atom distal to the carboxyl group of the double bond.^7^, ^8^ n‐3 PUFAs are mainly present in fish oil. These are essential fatty acids for the human brain and immune system and can only be obtained from diet.^9^, ^10^, ^11^

Previous studies have shown that high doses of n‐3 PUFA reduce serum lipid levels, which may further reduce the risk of cardiovascular disease.^12^, ^13^, ^14^ A number of studies have shown that regularly consume fish or take supplements containing n‐3 fatty acids can reduce the incidence of CVD.^15^, ^16^ However, some studies have found that additional supplementation of n‐3 PUFAs was not significantly associated with reduced CVD, suggesting that n‐3 PUFA supplements use should not be recommended for people at high risk of.^17^, ^18^ REDUCE‐IT exclusively used eicosapentaenoic acid (EPA) at very high doses (4 g/day),^16^ while STRENGHT used a combination of EPA and docosahexaenoic acid at lower doses (1.5 g/day),^17^ and the two groups were given different placebos, may making the results consistent. Another study that followed a large population for about 6.2 years also found that when compared with the placebo group, supplementation of n‐3 PUFAs did not reduce the incidence of CVD.^19^ The issue of n‐3 PUFA in atherosclerosis is controversial, and the effect of n‐3 PUFAs on CVD needs to be further explored.

Rabbits are commonly used to study atherosclerosis. Rabbits have more similarities with humans, their blood vessels are larger, and it is easier to conduct experimental operations on rabbits.^20^, ^21^ In addition, the lipoprotein profiles and lipid metabolism of rabbits are similar to those of humans, and the main lipoproteins in both are low‐density lipoprotein, which is more suitable for studying the correlation between atherosclerosis and lipoproteins.^21^, ^22^ Rabbits are more sensitive to a high‐cholesterol diet (HCD), are prone to hypercholesterolemia and atherosclerotic lesions, and are more likely to have increased levels of total cholesterol (TC) and low‐density lipoprotein cholesterol (LDL‐C).^22^, ^23^, ^24^, ^25^ Comparing the characteristics of rabbits and human atherosclerotic lesions in basic pathology and pathophysiology, it was found that the lesions in rabbits and humans were relatively similar.^26^, ^27^

The *Fat*‐*1* gene is a key gene for the synthesis of n‐3 PUFAs and was first identified in *Caenorhabditis elegans*. The *Fat*‐*1* gene can encode n‐3 fatty acid desaturase and catalyses the third carbon atom at the methyl end of the carbon chain in the substrate n‐6 PUFA to form an unsaturated double bond to form n‐3 PUFAs.^10^, ^28^ *Fat*‐*1* transgenic rabbits that overexpress n‐3 PUFAs have been used to study the relationship between n‐3 PUFAs and atherosclerosis.

## 2 MATERIALS AND METHODS

### 2.1 Animals

*Fat*‐*1* sequences were obtained from the *C*.* elegans* cDNA. Transgenic rabbits were created by overexpressing n‐3 PUFAs under control promoter SV40 by cloning the *Fat*‐*1* gene into the pIRES2‐AcGFP1 vector to construct the pIRES2‐AcGFP1‐△15 vector. The transgenic rabbits containing the *Fat*‐*1* gene were then obtained by testicular injection.

In the testicular direct injection method, exogenous DNA is wrapped in liposomes, and the liposome/DNA complex is punctured by injection, which then penetrates into the convoluted tubules of the testis through a wound or body fluid circulation and osmosis. This led to the transfection of germ cells. Exogenous DNA directly injected into the testis can be transfected into spermatogenic cells at all levels, and the sperm membrane can fuse liposomes, sperm cells absorb exogenous DNA and a large number of transgenic sperms can be obtained through spermatogenic cell proliferation and differentiation. Transgenic rabbits can then be produced by natural mating. The Fat‐1 construct and genotyping used in this study was created as described previously.^29^

A total of 24 male transgenic rabbits of *Fat*‐*1* (*n* = 12) and their littermates (WT) (*n* = 12), weighing 2.5 ± 0.5 kg and aged 3 months, were kept under standardized conditions (light of the feeding room was kept under a 12 h light/dark cycle, temperature 20–24°C, relative humidity 40%–70% and free access to food and water). Transgenic and WT rabbits were fed an HCD containing 0.3% cholesterol and 3% soybean oil for 16 weeks to induce atherosclerotic lesions. The animal study protocol was approved by the Laboratory Animal Administration Committee of the Xi'an Jiaotong University, and the guidelines were based on the Laboratory Animal Guidelines of the Xi'an Jiaotong University and the Guide for the Care and Use of Laboratory Animals published by the US National Institutes of Health (NIH Publication No.85‐23, Revised 2011).

### 2.2 Fatty acids analysis

The total fatty acids of livers and muscles of the two groups were extracted with benzene and petroleum ether at a ratio of 1:1 (v/v). Adding KOH‐methanol solution for methyl ester. The fatty acid compositions were determined using gas chromatography (GC) (Shimadzu Emit Co., Ltd.), and identified fatty acids were presented as area percentage of total fatty acids.

### 2.3 Plasma lipids analysis

Blood samples were collected from the middle ear artery of the rabbits once every two weeks. Approximately 2 ml blood samples containing EDTA anticoagulant were collected and centrifuged at 825 *g* for 15 min at 4°C. Triglyceride (TG), TC, high‐density lipoprotein cholesterol (HDL‐C) and LDL‐C levels were measured in each group using BioSino assay kits (Bio‐Technology & Science, Inc.).^30^ Plasma levels of tumour necrosis factor‐α (TNF‐α) and interleukin‐6 (IL‐6) were measured using ELISA kits (R&D Systems).

### 2.4 Glucose tolerance test and insulin tolerance test

Glucose tolerance test (GTT) was conducted after a 16 h fast. Glucose (0.6 g/kg body weight) was injected through the ear vein and blood samples are collected through the ear artery at 0, 5, 10, 15, 20, 30, 45, 60, 75 and 120 min. Insulin tolerance test (ITT) was also conducted after a 16 h fasting, and rabbits received an ear vein injection of 1.0 U/kg body weight insulin. Blood samples were collected 0, 5, 10, 15, 20, 30, 45, 60, 75 and 120 min after injection. Blood glucose was measured using a glucometer (Johnson & Johnson One Touch Ultra) and the plasma insulin assay was conducted using an ELISA insulin kit (Yanaihara Institute Inc.). The Insulin resistance index (IR‐ index) was calculated as described previously.^31^

### 2.5 Atherosclerotic lesions analysis

At the end of HCD feeding, all rabbits were euthanized. The whole arterial aorta was collected, opened longitudinally and fixed in 10% neutral buffered formalin. Then, the aorta was stained with Sudan IV, and the *en face* area of atherosclerotic lesions was quantified. To analyse the histological lesions, the aortic arch was cut into 8–10 sections, embedded in paraffin and cut into 4 µm thick serial sections, as described previously.^30^ Sections were stained with haematoxylin and eosin (H&E) and Elastica van Gieson (EVG) staining. The lesion composition of the atherosclerosis plaque was evaluated after immunostaining with mAbs against α‐smooth muscle actin (HHF35) (1:200; Dako) and macrophage (RAM11) (1:200; Dako) as previously described.^30^ The lesion area was measured using WinROOF 6.5 (Mitani Co.).

### 2.6 TUNEL assay

Apoptotic cells in aortic arch were detected using One‐Step TUNEL Apoptosis Assay Kit (Beyotime Biotechnology) as previously described.^32^

### 2.7 Statistical analysis

The results were analysed statistically by *t* test using GraphPad Prism 8.0. Results are presented as mean ± SEM. Statistical significance was set as *p < 0.05.

## 3 RESULTS

### 3.1 Expression level of fatty acids in Fat‐1 transgenic rabbits

In this study, genotypes of rabbits were identified by agarose gel electrophoresis (Figure S1A). Then, GC was used to measure changes in fatty acids in transgenic meat rabbits. The ordinate is the peak area, and the ordinate is the retention time of various fatty acids. The types and relative percentages of fatty acids were determined using GC (Figure S2A). Fatty acid levels in liver of the rabbits were analysed. Compared with the WT group, the level of total n‐6 PUFAs in the liver of the Fat‐1 group was decreased and total n‐3 PUFAs increased. The ratio of n‐6/ n‐3 in liver in the Fat‐1 group was significantly lower than that in the WT group (Table 1). The n‐6 PUFAs decreased and n‐3 PUFAs increased in the livers of Fat‐1 transgenic rabbits. The ratio of n‐6/ n‐3 in muscle in the Fat‐1 group was also significantly lower than that in the WT group (Table 2). We also measured the fatty acid composition in plasma of transgenic rabbits and control rabbits after weaning. We found that the C20:5n‐3 of transgenic rabbits was lower than that of the control group, and the difference was extremely significant (*p* < 0.01) (Table S1). These results suggest that Fat‐1 plays a key role in the synthesis of n‐3 PUFAs by using n‐6 PUFA as a substrate in transgenic rabbits, and the preparation of Fat‐1 transgenic rabbits was successful.

**TABLE 1:** Fatty acid compositions (%) of the livers in *Fat*‐*1* transgenic and control rabbits

Fatty acids	Control	Fat‐1
C18:2n‐6	20.46 ± 1.24	5.62 ± 0.39**
C18:3n‐6	1.31 ± 0.23	3.29 ± 0.47**
C20:4n‐6	5.88 ± 0.39	4.62 ± 0.23*
C22:4n‐6	1.81 ± 0.11	2.01 ± 0.15
C18:3n‐3	0.53 ± 0.04	2.3 ± 0.12**
C20:5n‐3	1.21 ± 0.17	1.82 ± 0.22*
C22:5n‐3	0.19 ± 0.03	1.34 ± 0.4**
C22:6n‐3	0.61 ± 0.05	0.71 ± 0.02
SUMn‐6	29.46 ± 1.46	15.55 ± 0.53**
SUMn‐3	2.47 ± 0.18	4.75 ± 0.25**
n‐6/n‐3	13.19 ± 1.14	3.35 ± 0.14**

Note

Data are expressed as mean ± SEM. *n* = 12 for each group.

*p < 0.05, **p < 0.01.

**TABLE 2:** Fatty acid compositions (%) of the muscle in *Fat*‐*1* transgenic and control rabbits

Fatty acids	Control	Fat‐1
C18:2n‐6	19.62 ± 1.43	8.88 ± 1.93**
C18:3n‐6	1.15 ± 0.3	1.35 ± 0.18
C20:4n‐6	4.43 ± 0.81	6.2 ± 0.65
C22:4n‐6	1.21 ± 0.12	2.22 ± 0.13**
C18:3n‐3	1.07 ± 0.09	3.96 ± 1.54
C20:5n‐3	1.13 ± 0.2	2.35 ± 0.2**
C22:5n‐3	0.15 ± 0.03	1.31 ± 0.39**
C22:6n‐3	0.51 ± 0.08	0.9 ± 0.09**
SUMn‐6	26.11 ± 1.38	18.39 ± 2.04**
SUMn‐3	2.54 ± 0.15	7.05 ± 1.55**
n‐6/n‐3	10.96 ± 0.89	3.43 ± 0.73**

Note

Data are expressed as mean ± SEM. *n* = 12 for each group.

**p < 0.01.

### 3.2 The effect of n‐3 PUFA overexpression on plasma lipid

**FIGURE 1:**
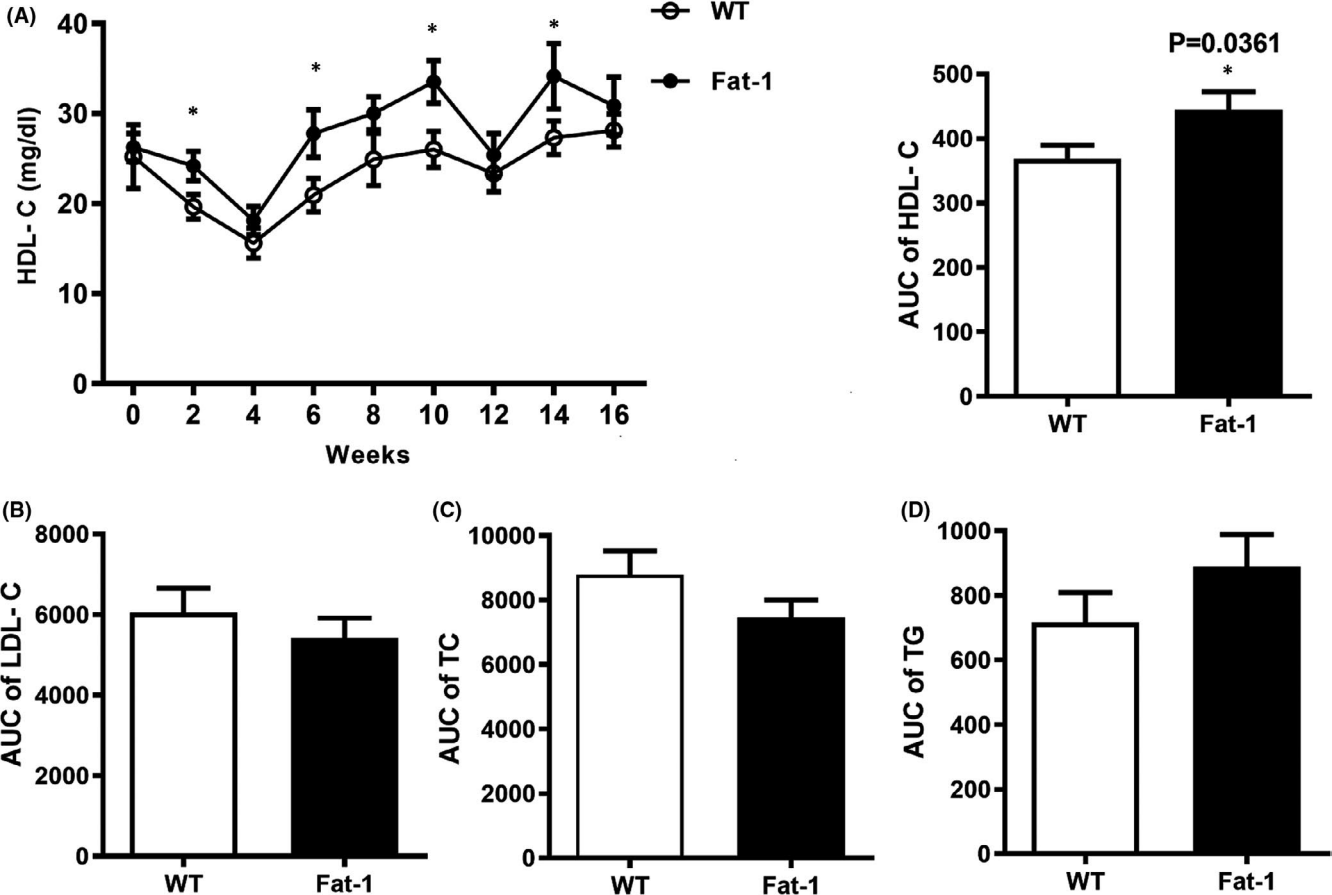
Plasma lipids of the rabbits. (A) High‐density lipoprotein cholesterol (HDL‐C) levels and the area under curve (AUC) (*p* = 0.0361). (B) Low‐density lipoprotein cholesterol (LDL‐C) (*p* = 0.4314). (C) Total cholesterol (TC) (*p* = 0.1694). (D) Triglyceride (TG) (*p* = 0.2195). Data are expressed as the mean ± SEM. *n* = 12 for each group

The plasma lipid levels in rabbits were measured every two weeks. In order to systematically analyse the effect of n‐3 PUFA overexpression on plasma lipids during the entire experiment, we calculated the incremental area under curve (AUC) according to the trapezoidal rule.^31^ We found that the expression of *Fat*‐*1* significantly increased the plasma HDL‐C levels (*p* < 0.05, Figure 1A), but the plasma LDL‐C, TC and TG levels showed no significant difference between the two groups (*p* > 0.05, Figure 1B–D). These results suggest that *Fat*‐*1* transgenic plants can affect the level of HDL‐C.

The body weight of rabbits was tested every two weeks during the study, but no significant difference was found between the transgenic and WT groups (*p* > 0.05, Figure S3A). The main organs of rabbits were weighed, and no significant difference was observed in the weight of the major organs between the two groups (*p* > 0.05, Figure S3B). Plasma IL‐6 and TNF‐α levels were measured in rabbits. However, there was no difference between the two groups (*p* > 0.05, Figure S4A,B), suggesting that overexpression of n‐3 PUFA does not affect the expression level of inflammatory cytokines in rabbits.

### 3.3 n‐3 PUFA overexpression alleviates atherosclerotic lesions

**FIGURE 2:**
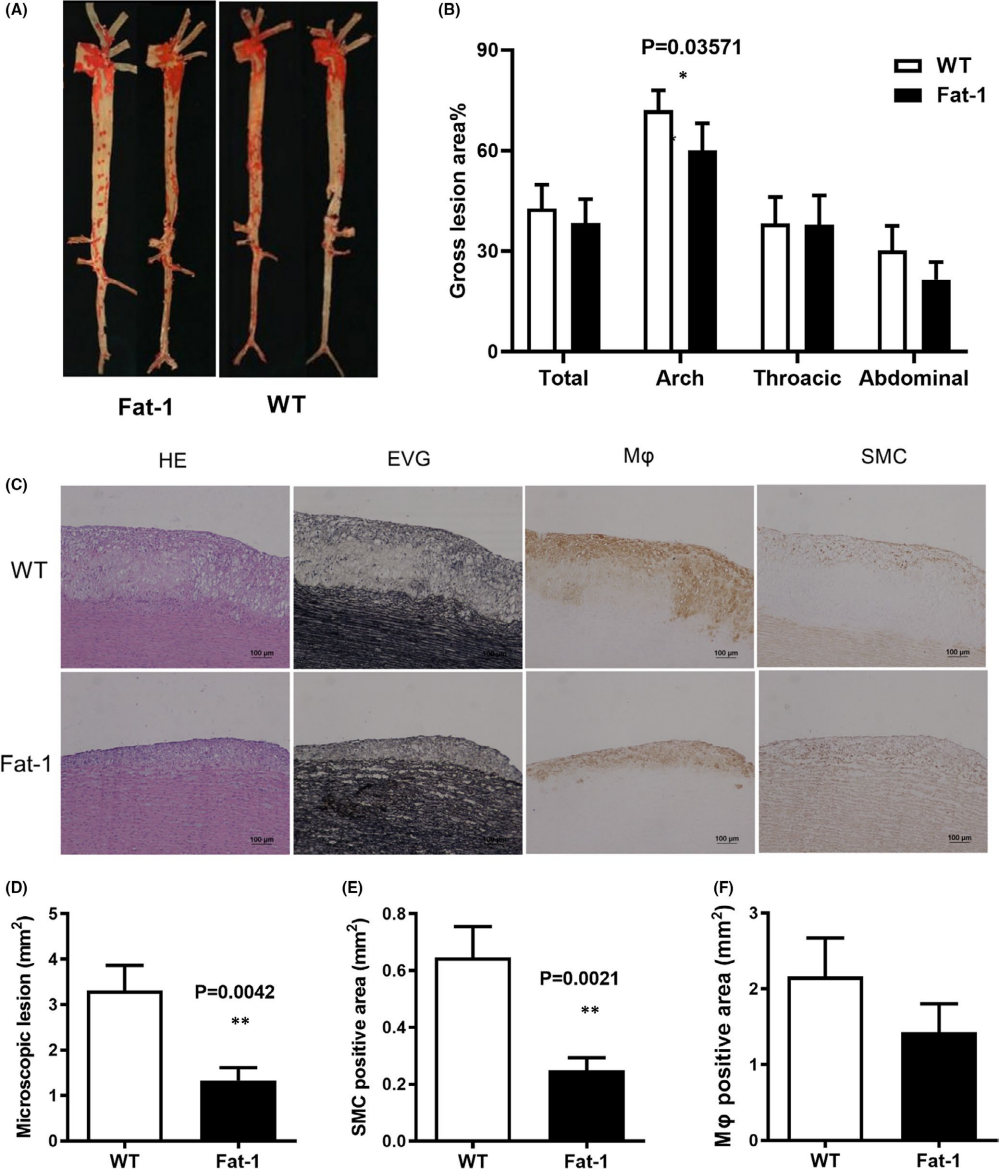
Atherosclerotic lesions analysis. (A) Representative Sudan IV staining images of aortas in the rabbits fed with high‐cholesterol diet. (B) Quantification of the total aorta, aortic arch, and thoracic and abdominal aorta lesion areas (%) (*p* = 0.662792, *p* = 0.03571, *p* = 0.979666, *p* = 0.342861). (C) Aortic arch section haematoxylin and eosin (H&E) staining, Elastica van Gieson (EVG) staining and macrophages and smooth muscle cells (SMC) immunohistochemical staining. (D) Quantitative analysis of lesion area (*p* = 0.0042). (E) The positive area of SMC (*p* = 0.0021). (F) The positive area of macrophages (Mφ) (*p* = 0.2034). Data are expressed as mean ± SEM. *n* = 12 for each group

At the end of HCD feeding, all rabbits were euthanized. The aorta of rabbits was collected for pathological and histological analyses. Figure 2A shows representative images of rabbit aorta stained with Sudan IV. An *en face* analysis of the atherosclerotic lesion area suggested that the lesion area of the aortic arch was significantly reduced in the *Fat*‐*1* group (*p* < 0.05), and the lesion areas of the total aorta, thoracic aorta and abdominal aorta were statistically similar between the groups (*p* > 0.05; Figure 2B).

To further analyse the histological characteristics of atherosclerotic lesions, we sectioned the aortic arch and analysed the lesion features by HE staining, EVG staining, macrophages and SMC immunohistochemical staining. It was found that the lesions were mainly present in the fatty streak lesion stage (Figure 2C). Microscopic analysis was used to quantify the aortic arch lesions in EVG‐stained sections. The results showed that n‐3 PUFA overexpression reduced atherosclerotic lesion area (*p* < 0.01, Figure 2C,D). Compared with the WT group, the number of SMCs in lesions was significantly reduced in the *Fat*‐*1* group (*p* < 0.01, Figure 2E). However, there was no difference in the number of macrophages in the lesions between the two groups (*p* > 0.05, Figure 2F).

We found that there was no difference in apoptosis at the aortic arch lesions between the two groups of rabbits (Figure S5).

### 3.4 The effect of n‐3 PUFA overexpression on glucose and insulin sensitivity

**FIGURE 3:**
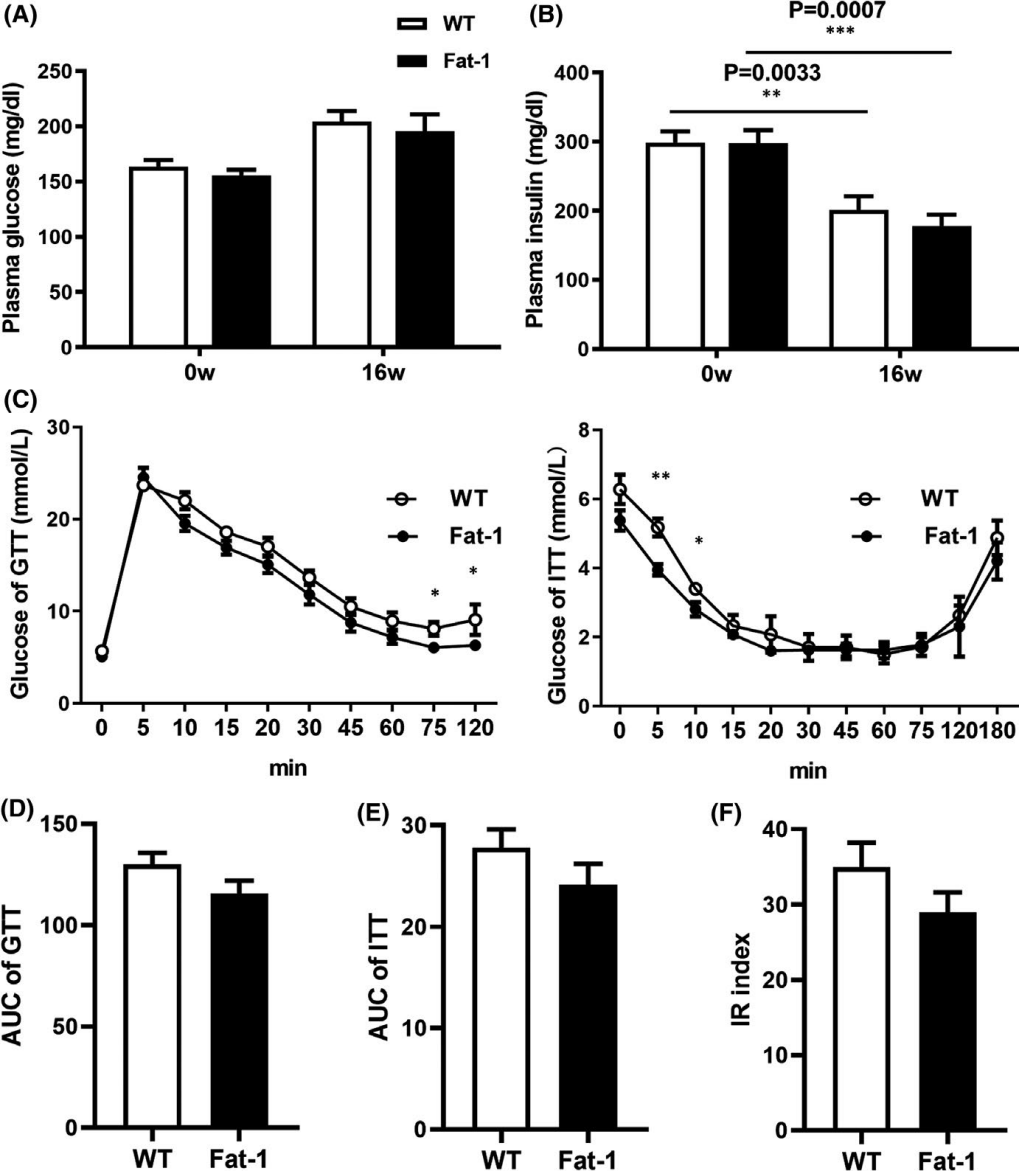
Glucose and insulin levels. (A) Blood glucose level (*p* = 0.336626, *p* = 0.638573). (B) Insulin level (*p* = 0.0033, *p* = 0.0007). (C) Blood glucose levels during glucose tolerance test (GTT) and blood glucose levels during Insulin tolerance test (ITT). (D) AUC of GTT (*p* = 0.1324). (E) AUC of ITT (*p* = 0.2313). (F) Insulin resistance index (IR index) (*p* = 0.2022). Data are expressed as mean ± SEM. *n* = 12 for each group. *p < 0.05, **p < 0.01

Glucose and insulin levels were measured in the *Fat*‐*1* and WT rabbit groups during HCD feeding. The plasma glucose of rabbits at 0 and 16 weeks did not show significant differences *p* > 0.05, Figure 3A). The plasma insulin levels showed there are significant difference between 0 and 16 weeks (*p* < 0.05), but there was no difference between the *Fat*‐*1* group and WT group (*p* > 0.05, Figure 3B). As shown in Figure 3C, after glucose injection, there was no significant difference between the two groups at 0–75 min (*p* > 0.05). During 75–120 min, the plasma glucose level of the *Fat*‐*1* group was significantly lower than that of the WT group (*p* < 0.05, Figure 3C left). The ITT results showed that after injection of insulin, the plasma glucose of the *Fat*‐*1* group was significantly different between the two groups from 5 to 10 min (*p* < 0.05, Figure 3C right). To explore the overall effect of n‐3 PUFA overexpression on glucose tolerance, the AUC of plasma glucose was calculated, which suggested that the plasma glucose levels of the two groups were not significantly different (*p* > 0.05, Figure 3D). According to the AUC of ITT, the plasma glucose levels of the two groups were not significantly different (*p* > 0.05, Figure 3E). To explore the effect of n‐3 PUFA overexpression on insulin resistance (IR), the IR of rabbits was calculated after GTT and ITT, and no significant difference was observed in insulin sensitivity between the two groups (*p* > 0.05, Figure 3F). These results indicated that n‐3 PUFA overexpression did not affect glucose metabolism in rabbits.

## 4 DISCUSSION

Cardiovascular disease and subsequent ischaemic complications, including myocardial infarction and stroke, are the most common causes of morbidity and mortality.^33^, ^34^ The underlying cause of most vascular diseases is atherosclerosis, a chronic disease characterized by lipid accumulation in the intima that progresses asymptomatically for years. Rabbits have unique advantages as an animal model of atherosclerosis. The rabbits were fed HCD to induce atherosclerosis lesions and the effects of HCD on cardiovascular disease in rabbits or transgenic rabbits were observed.^21^, ^22^, ^23^, ^25^, ^30^, ^31^ Therefore, rabbits are used in current study.

*Fat*‐*1* is a key gene in the synthesis of n‐3 PUFAs and converts n‐6 PUFAs to n‐3 PUFAs using n‐3 desaturase. The results of fatty acid content showed that the level of total n‐6 PUFA in the *Fat*‐*1* group was decreased and total n‐3 PUFAs were increased in the liver and muscle. This suggested that the n‐3 PUFAs in *Fat*‐*1* transgenic rabbits were overexpressed, and the preparation of *Fat*‐*1* transgenic rabbits was successful. In this study, we explored the role of n‐3 PUFAs in atherosclerosis. After 16 weeks of continuous HCD feeding, n‐3 PUFA overexpression had no significant effect on body weight or organ weight in the two groups. Compared with the WT group, the plasma HDL‐C level of rabbits in the *Fat*‐*1* group was significantly higher. HDL‐C is a small and dense substance in the blood that is rich in a variety of lipid and protein macromolecules and is involved in the reverse transport of cholesterol to improve atherosclerosis.^35^

As the results showed that HDL‐C was elevated in transgenic rabbits, we think that n‐3 PUFAs can increase plasma HDL‐C and modify the expression of key genes and proteins involved in reverse transport of cholesterol. A growing body of research suggests an inverse relationship between HDL‐C levels and cardiovascular risk. Together, these findings demonstrate that focusing on HDL‐C offers a new and additional strategy for reducing cardiovascular risk.^36^ One study reported that the addition of HDL‐C may not only inhibit progression of atherosclerosis but also reduce established atherosclerotic lesions by HDL‐C administration.^37^ Thus, we suggest that the reduction of atherosclerotic lesions by n‐3 PUFAs may be related to increased HDL‐C levels. The protective effect of HDL on atherosclerosis depends not only on HDL concentration (quantity) but also on HDL function (quality).^38^, ^39^ The benefit of n‐3 PUFAs may be due to the improved HDL function. Trials have shown that increased HDL antioxidant capacity is associated with a reduced risk of atherosclerosis, and certain HDL subspecies can act as natural antioxidants, preventing lipid oxidation on LDL. Its antioxidant function may be due to the inhibition of the synthesis of free radicals and reactive oxygen species by HDL lipids and related enzymes, or the transfer of oxidation‐susceptible lipids from LDL and biofilms to HDL catabolism.^40^ Several nutritional interventions to improve the antioxidant activity of HDL have been proposed, but with limited success.^41^, ^42^ Therefore, we think that the benefit of n‐3 PUFAs may be due to increased HDL‐C levels and improved HDL function. In future studies, we will evaluate the function of HDL and further explore the role of HDL in atherosclerosis.

In our study, *en face* staining of the aorta and histological analysis showed that n‐3 PUFA overexpression significantly reduced atherosclerotic lesions in *Fat*‐*1* transgenic rabbits, and there was a significant reduction atherosclerotic plaque in the aortic arch, histological lesions and the number of SMCs in lesions. n‐3 PUFAs may reduce SMC migration and alter phenotypes through multiple mechanisms, this increases its potential benefits for atherosclerosis. It was found that n‐3 PUFAs reduced SMC migration by inhibiting uPAR expression and regulated the MEK/ERK signalling pathway to affect its expression and induce phenotypic changes in SMC.^43^ The function of SMC on atherosclerosis in Fat‐1 transgenic rabbit needs to be further studied in the future.

However, there are several limitations in the current study. First, it is not known whether n‐3 PUFAs delay the progression of atherosclerosis by increasing in plasma HDL‐C and decreasing the amount of SMC in atherosclerotic plaques. Second, we did not detect directly the expression levels of Fat‐1 mRNA and protein in the different tissues. Third, we did not measure the levels of n‐6 PUFA and n‐3 PUFA in a high‐cholesterol diet. Furthermore, this study is a preliminary exploration, and we have not measured the stability of atherosclerotic lesions. The role and mechanism of n‐3 PUFA in atherosclerosis need further studies.

In conclusion, endogenously synthesized n‐3 PUFAs can inhibit atherosclerosis in *Fat*‐*1* transgenic rabbits, and this process may depend on the increase in plasma HDL‐C and the decrease in the amount of SMC in atherosclerotic plaques. These results suggest that n‐3 PUFAs may help prevent CVD.

## CONFLICTS OF INTEREST

The authors declare no conflict of interest.

## AUTHOR CONTRIBUTIONS

**Chenyang Zhang:** Conceptualization (equal); Methodology (equal); Visualization (equal); Writing – original draft (lead). **Xiaojing Wang:** Conceptualization (equal); Methodology (lead); Resources (equal); Visualization (equal). **Suping Sun:** Methodology (supporting); Resources (equal); Visualization (supporting). **Yu Fu:** Data curation (equal); Software (supporting). **Yi Wu:** Conceptualization (equal); Visualization (equal); Writing – review & editing (supporting). **Sihai Zhao:** Conceptualization (supporting); Methodology (supporting); Writing – review & editing (supporting). **Xinzhong Fan:** Conceptualization (supporting); Methodology (supporting); Supervision (supporting); Writing – review & editing (supporting). **Enqi Liu:** Conceptualization (equal); Funding acquisition (lead); Methodology (equal); Supervision (lead); Writing – original draft (equal); Writing – review & editing (lead).

## Supporting information

Supplementary Material

## Data Availability

All data generated or used during this study are available from the corresponding authors, Enqi Liu or Xinzhong Fan, upon reasonable request.
