# Supplementary material for: Fat‐1 expression alleviates atherosclerosis in transgenic rabbits
Source: J Cell Mol Med. 2022 Jan 18;26(4):1306–14. doi: 10.1111/jcmm.17188 (PMC8831984; doi:10.1111/jcmm.17188)
Supplement: Supplementary file 1 — Supplementary Material [file JCMM-26-1306-s001.docx]

**Supplementary materials**

**Supplementary Table 1.** Fatty acid compositions (%) of plasma in *Fat-1* transgenic and control rabbits

| Fatty Acids | control | Fat-1 |
| --- | --- | --- |
| C18:2n-6 | 6.7±1.66 | 10.08±2.95 |
| C20:4n-6 | 1.55±0.29 | 1.49±0.24 |
| C18:3n-3 | 0.51±0.05 | 0.83±0.15 |
| C20:5n-3 | 0.65±0.05 | 0.34±0.06** |
| C22:5n-3 | 0.16±0.01 | 0.20±0.00 |
| SUMn-6 | 8.24±0.38 | 11.57±2.85 |
| SUMn-3 | 1.27±0.14 | 1.22±0.14 |
| n-6/n-3 | 6.55±0.92 | 9.40±2.04 |

Data are expressed as mean ± SEM. n = 12 for each group. ** *P* < 0.01.


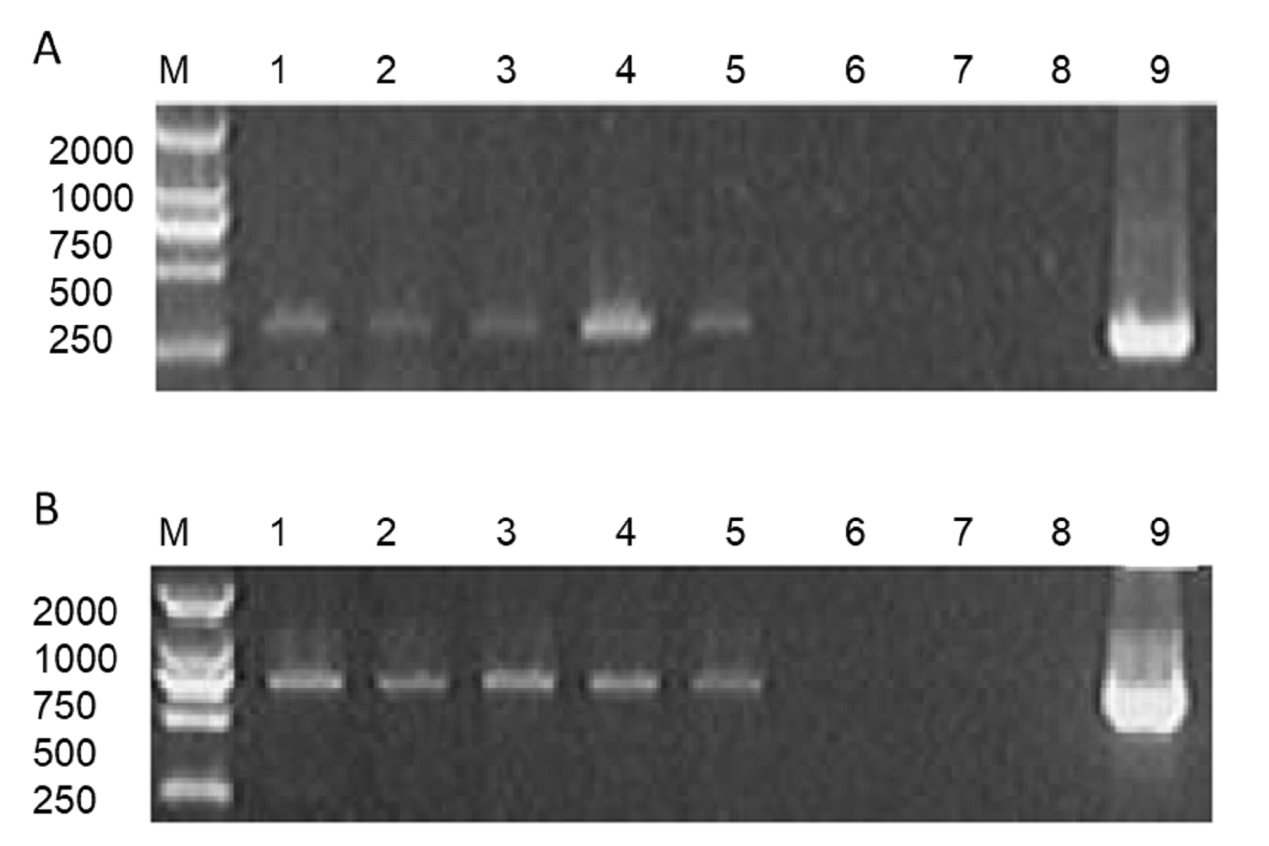


**Supplementary Figure 1.** Genotyping identification of transgenic rabbits. Lane 1-5 were positive samples, lane 6-7 were non- transgenic samples, lane 8 were blank control samples, and lane 9 were positive control samples. (A) Detection of the *Fat-1* gene. (B) Detection of the AcGFP1.

**
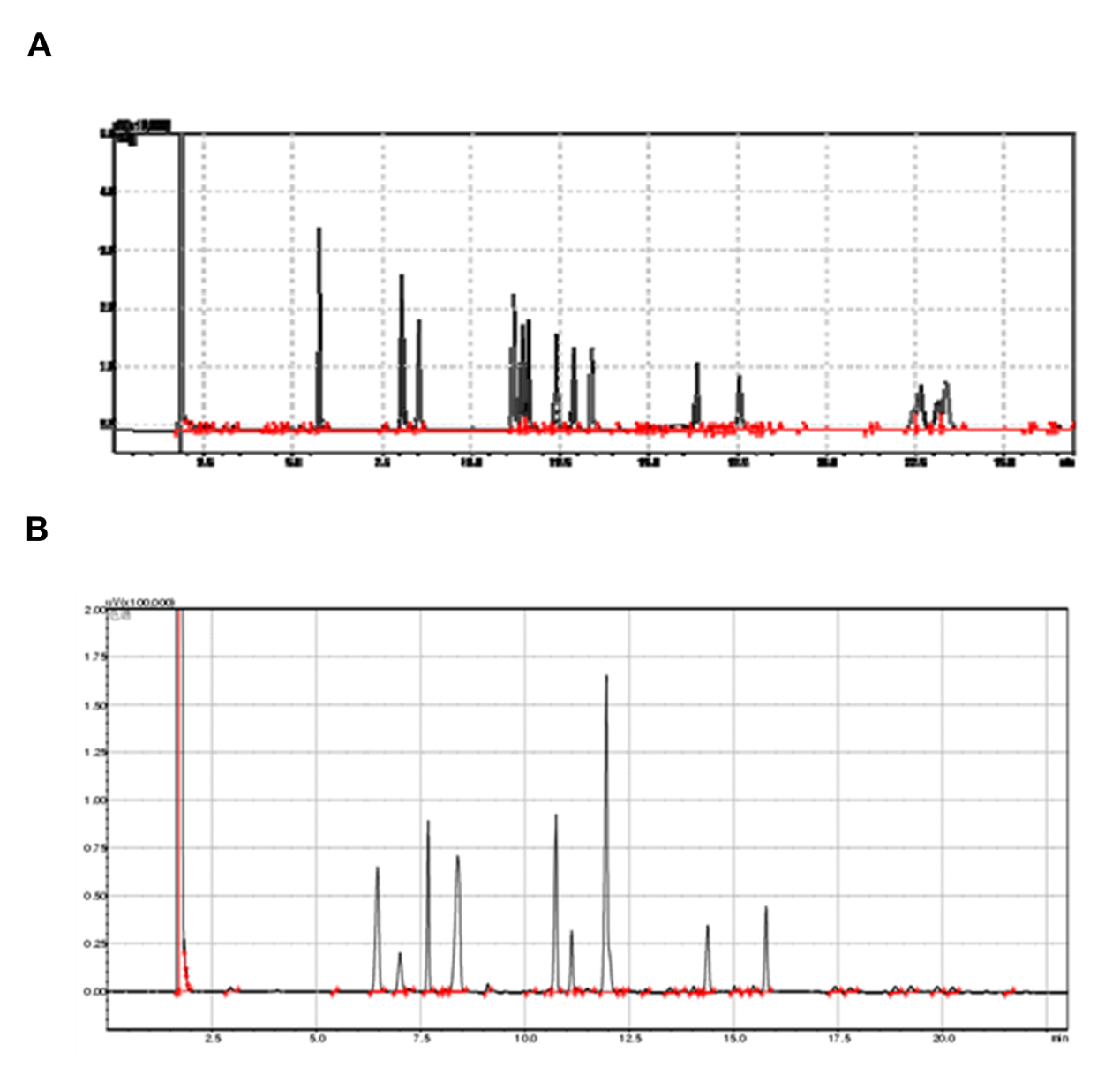
Supplementary Figure 2.** Fatty acid methylesters product time and peak. (A) standard product. (B) Sample.


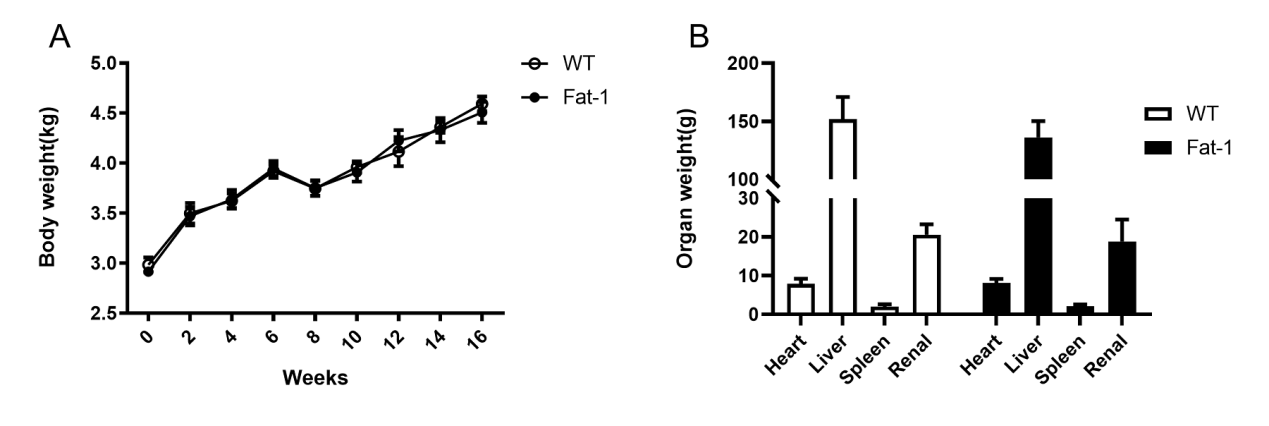


**Supplementary Figure 3.** (A) Body weight of rabbits. (B) Organ weight.

Data are expressed as mean ± SEM. n = 12 for each group.


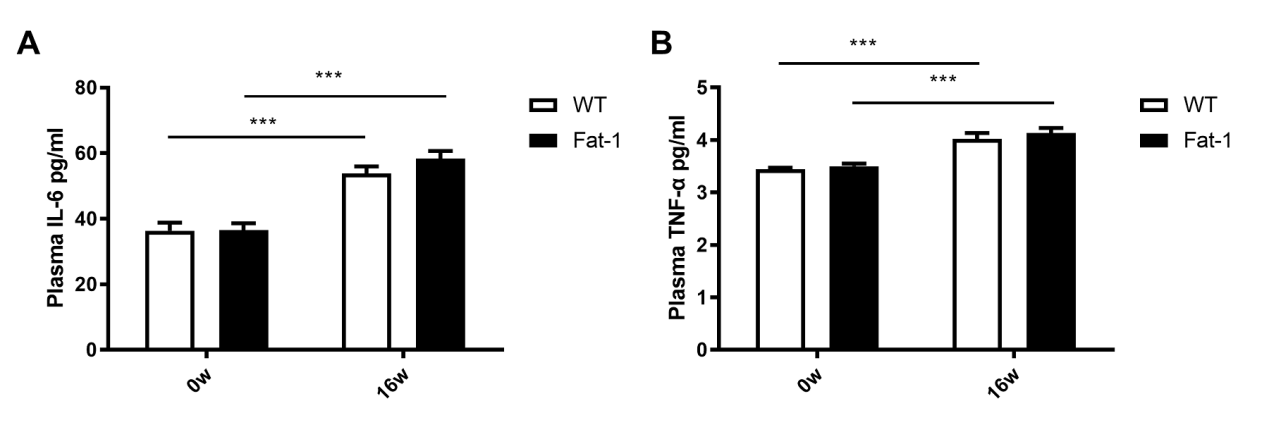


**Supplementary Figure 4.** (A) Plasma IL-6 in rabbits. (B) Plasma TNF-αlevel.

Data are expressed as mean ±SEM. n = 12 for each group. *** *P* < 0.001.


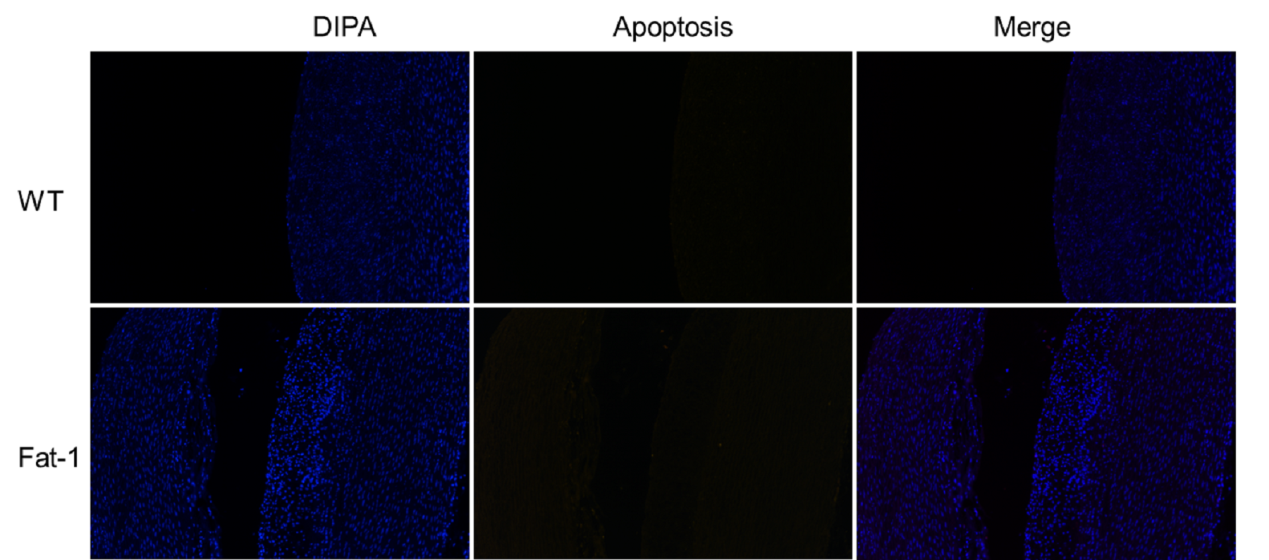


**Supplementary Figure 5.** Apoptosis in aortic lesions. n=5, for each group.
